# Prognostic Significance of Thrombomodulin mRNA in High‐Grade Soft Tissue Sarcomas after 10 years

**DOI:** 10.1111/os.12779

**Published:** 2020-10-04

**Authors:** Kunihiro Asanuma, Tomoki Nakamura, Yumiko Asanuma, Takayuki Okamoto, Takuya Kakimoto, Yuki Yada, Tomohito Hagi, Kouji Kita, Koichi Nakamura, Akihiko Matsumine, Akihiro Sudo

**Affiliations:** ^1^ Department of Orthopedic Surgery Mie University School of Medicine Tsu City Japan; ^2^ Department of Pharmacology, Faculty of Medicine Shimane University Izumo Japan; ^3^ Department of Orthopedic Surgery, Faculty of Medical Sciences University of Fukui Eiheiji Japan

**Keywords:** Metastasis, mRNA, Prognosis, Soft tissue sarcoma, Soft tissue tumor, Thrombomodulin

## Abstract

**Objective:**

To elucidate the correlation between expression of thrombomodulin (TM) mRNA from 83 benign soft tissue tumors or soft tissue sarcomas (STS) and clinicopathological parameters and to analyze the outcome of high‐grade STS patients after 10 years.

**Methods:**

Total RNA was extracted from 83 primary soft tissue tumors (15 benign tumors, 68 STS). TM mRNA normalized to glyceraldehyde‐3‐phosphate dehydrogenase was measured with real‐time quantitative polymerase chain reaction and compared to various clinicopathological parameters. The log‐rank test and Cox proportional hazard analysis were used to evaluate recurrence‐free survival, metastasis‐free survival, and overall survival.

**Results:**

Thrombomodulin mRNA levels were not significantly different between benign tumors and STS. In STS, TM mRNA levels were not significantly different between histologically high‐grade (*n* = 57) and low‐grade (*n* = 11) tumors. Following analysis of high‐grade STS at the 10‐year follow‐up, 21 patients had experienced a recurrence, 22 patients had experienced metastasis, and 23 patients had died of disease (DOD). TM levels were significantly higher in patients with metastasis or DOD patients. Receiver operating characteristic analysis for identifying 5‐year and 10‐year DOD determined the threshold for best sensitivity and specificity as 0.283. We divided patients into those with high (<0.283) and low (≤0.283) TM mRNA levels. Based on Kaplan–Meier analysis, a significant difference between the two groups was seen for recurrence‐free survival (5 years: low = 76.6%, high = 53.1%, 10 years: low: 67.0%, high 39.8%, *P* = 0.0122) and metastasis‐free survival (5 years: low = 86.3%, high = 40.2%, 10 years: low: 73.3%, high: 35.2%, *P* = 0.00023). Furthermore, the high TM group showed significantly worse prognosis than the low TM group (5 years: low = 90.1%, high = 42.3%, 10 years: low: 76.4%, high 31.3%, *P* = 0.00031). Thus, high levels of TM mRNA are associated with highly recurrent and metastatic potential and lead to poor prognosis. In multivariate Cox proportional hazard analysis, only high TM showed a significant difference in metastasis‐free survival (hazard ratio: 4.33, 95% confidence interval 1.61–11.6, *P* = 0.00359) and overall survival (hazard ratio: 3.69, 95% confidence interval 1.49–10.5, *P* = 0.00569).

**Conclusion:**

High levels of TM mRNA may be a significant predictor of recurrence, metastasis, and a poor outcome in STS patients after 10 years. TM is a candidate molecular marker and may be clinically useful for devising a therapeutic treatment strategy by prediction of prognosis.

## Graphical abstract

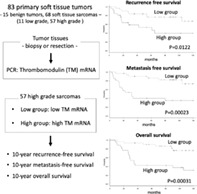

## Introduction

Thrombomodulin (TM, CD141), a transmembrane glycoprotein with 557 amino acids, comprises an NH_2_‐terminal lectin‐like region followed by epidermal growth factor‐like structures, an O‐glycosylation site‐rich domain, transmembrane domain, and cytoplasmic tail. TM plays an essential role as a receptor for thrombin. Thrombin‐containing complexes activate the anticoagulant pathway of protein C. Activated protein C proteolytically inactivates coagulation factors Va and VIIIa^1^, ^2^, ^3^.

Thrombomodulin is expressed on the cell surface of endothelial cells, monocytes, dendritic cells, neutrophils, platelets, megakaryocytes, smooth muscle cells, keratinocytes, meningeal cells, synovial lining cells, and mesothelial cells^4^, ^5^, ^6^, ^7^, ^8^, ^9^, ^10^. In addition, TM expression is reported in tumor cells such as non‐small cell lung cancer^11^, colorectal carcinoma^12^, bladder cancer^2^, and esophageal squamous cell carcinoma^13^. TM plays various important roles in tumor cell behavior. Experimentally, TM downregulates tumor cell proliferation^1^, ^2^, and TM mediates Ca^2+^‐dependent cell–cell adhesion^14^. Anti‐coagulation activity by TM decreases metastatic potential^15^. Clinically, absent or low expression of TM is associated with poor prognosis relative to high expression of TM in lung cancer^11^, breast cancer^16^, bladder cancer^2^, and colorectal carcinoma^12^. TM has a protective effect on tumor exacerbation, both experimentally and clinically. Although the function of TM in cancer cells and patients has been reported, the correlation between TM and soft tissue sarcomas (STS) is unclear. Contrary to reports about cancer and TM, we previously reported that a high level of soluble TM was a significant predictor of metastasis and poor prognosis in STS patients^17^. It is thought that the role of TM in tumor cells is different for cancer and STS. Therefore, we hypothesized that in contrast to cancer, an increase of TM mRNA levels is related to malignancy or exacerbation of recurrence, metastasis, or overall survival in STS patients.

The purpose of this study is: (i) to elucidate the correlation between expression of TM mRNA from 83 benign soft tissue tumors or STS and clinicopathological parameters; and (ii) to predict the outcome of high‐grade STS patients after 10 years.

## Materials and Methods

### *Patients*

Criteria for inclusion were: patients with primary soft tissue tumors who visited Mie University Hospital between 1982 and 2006 and who underwent complete tumor resection with a wide margin during the initial surgery. Criteria for exclusion were: patients with local recurrence or distant metastasis or who were referred for additional resection after inadequate resection in a previous hospital. A total of 83 patients were included in this study. The histopathological diagnosis was made by independent pathologists and classified according to the World Health Organization classification. Written, informed consent was obtained from each patient. For patients under the age of 19 years, informed consent was obtained from their parents or legal guardian. This study was approved by the Ethics Committee of the Mie University Graduate School of Medicine (approval number: 1310). All procedures performed in studies involving human participants were in accordance with the ethical standards of the Ethics Committee of Mie University and with the Helsinki Declaration.

### *Preparation of Tissue Samples*

All tumor tissue samples were obtained from patients who underwent surgical resection or an open biopsy at the Department of Orthopedic Surgery, Mie University Graduate School of Medicine. The tissue specimens were immediately collected and frozen in liquid nitrogen (for RNA extraction) or fixed for 24 h in 10% buffered formalin solution and embedded in paraffin (for histopathological analysis).

### *Quantitative Real‐Time Polymerase Chain Reaction*

Total RNA was extracted from each sample using ISOGEN (Nippon Gene, Tokyo, Japan), according to the manufacturer's instructions and then reverse‐transcribed into cDNA using the First Strand cDNA Synthesis Kit (Roche Applied Science, Mannheim, Germany). The TaqMan Gene Expression Master Mix and the TaqMan Gene Expression Assay (Applied Biosystems, Foster City, CA, USA) were used to quantitatively analyze the expression of glyceraldehyde‐3‐phosphate dehydrogenase (GAPDH) and TM. GAPDH was used as an endogenous housekeeping gene for normalization. Real‐time quantitative polymerase chain reaction amplifications were performed using an ABI PRISM 7000 Sequence Detection System (Applied Biosystems). Standard curves were generated using cDNA samples from HeLa cells. The relative expression levels of each target gene were indicated by calculating the ratio to the expression levels in the HeLa cells. All assays were performed in triplicate.

### *Statistical Analysis*

Clinicopathological analysis was performed to compare the TM mRNA levels to various clinical parameters using the Mann–Whitney *U‐*test for quantitative data. To evaluate the threshold for detecting mortality due to disease, receiver operating characteristic (ROC) curves were generated. ROC curves were created by plotting the sensitivity on the y‐axis and the false positive rate (1 − specificity) on the x‐axis. To measure the effectiveness of the TM level as a marker for mortality due to disease, the area under the curve (AUC) was assessed. Local recurrence‐free survival (RFS) was defined as the time from the initial treatment to the date of clinically documented local recurrence. Metastasis‐free survival (MFS) was defined as the time from the initial treatment to the date of clinically documented distant metastasis. Overall survival (OS) was defined as the time from the initial treatment to the date of mortality attributed to the neoplasm. Kaplan–Meier survival plots and log‐rank tests were used to assess the differences in time to local recurrence, distant metastasis, or OS. To adjust for the imbalance in prognostic factors among patients, Cox proportional hazard analysis was used. *P* < 0.05 was considered statistically significant.

## Results

### *Patient Characteristics*

The present study involved 83 patients (51 males and 32 females). The average age was 50.1 years (range: 0–85 years). Histopathological diagnosis was as follows: 15 benign tumors, including 5 schwannomas, 3 lipomas, 3 neurofibromas, and 4 others; 68 STS, including 21 liposarcomas, 17 undifferentiated pleomorphic sarcomas (UPS), 9 synovial sarcomas, 7 malignant peripheral nerve sheath tumors, 4 clear cell sarcomas, and 10 others. The 57 high‐grade STS included 17 UPS, 11 liposarcomas, 9 synovial sarcomas, 7 malignant peripheral nerve sheath tumors, 4 clear cell sarcomas, and 9 others. In the benign soft tissue tumors and STS, TM mRNA levels were not significantly different between patients according to sex and malignancy. Patients over 55 years of age showed significantly higher TM values than those under 55 years (*P* = 0.00488) (Table 1). However, based on Spearman's rank correlation, age was only weakly correlated with TM mRNA levels (rho = 0.248, *P* = 0.0248). In addition, in STS, TM mRNA levels were not significantly different between histologically high‐grade (*n* = 57) and low‐grade (*n* = 11) tumors (Table 1). TM mRNA levels and characteristics of patients with high‐grade STS tumors are shown in Table 2. In high‐grade STS, tumors over 10 cm and radiotherapy group showed significantly higher TM values than those less than 10 cm.

**TABLE 1:** Characteristics of patients with benign soft tissue tumors and soft tissue sarcomas

Characteristics		N (83)	TM (median)	*P*‐value
Sex	Male	51	0.358	0.61
	Female	32	0.271	‐
Age	<55	44	0.227	0.00488*
	55≤	39	0.761	‐
Malignancy	Benign	15	0.363	0.766
	STS	68	0.288	‐
	Low	11	0.445	0.438
	high	57	0.256	‐

Sex, age, malignancy, and histological grade are listed and noted by number. Thrombomodulin (TM) mRNA levels were compared for each parameter using the Mann–Whitney test. *Age shows significant differences.

**TABLE 2:** Characteristics of patients with STS

Characteristics of high‐grade STS patients		*n* = 57	TM (median)	*P*‐value
Sex	Male	35	0.256	0.942
	Female	23	0.273	‐
Age	<55	33	0.169	0.0509
	55≤	24	0.842	‐
Tumor size	<10	36	0.219	0.0403*
	≤10	21	0.604	‐
Location	Extremity	41	0.301	0.993
	Trunk	16	0.172	‐
Tumor depth	Superficial	6	0.877	0.927
	Deep	51	0.259	‐
Chemotherapy	−	32	0.253	0.78
	+	25	0.282	‐
Radiotherapy	−	26	0.198	0.0473*
	+	31	0.378	‐

Only patients with histological high‐grade tumors were analyzed. Patients with low‐grade tumors were excluded. *Based on the Mann–Whitney *U*‐test, thrombomodulin (TM) values by tumor size and radiotherapy were significantly different. STS, soft tissue sarcomas.

### *Recurrence, Metastasis, and Survival of Patients with High‐Grade Soft Tissue Sarcomas*

The average follow‐up period for patients with STS was 85.7 months (range, 8–338 months). At the 5‐year observation time point, 18 patients had experienced a recurrence, 19 patients had experienced metastasis, and 18 patients had died of disease (DOD). Recurrence, metastasis, and DOD of the top three pathological types were shown. TM mRNA levels were significantly higher in the patients with metastasis or DOD patients than in patients without metastasis or patients who were alive. After an additional 5 years, three more patients experienced a recurrence, three more patients experienced metastasis, and five more patients had DOD. Similar to the 5‐year observation time point, mRNA levels were significantly higher in the patients with metastasis or DOD patients at the 10‐year observation time point (Table 3).

**TABLE 3:** Characteristics of patients with STS

Characteristics			Total	UPS	Liposarcoma	Synovial sarcoma	TM (median)	*P*‐value
n			57	17 (%)	11 (%)	9 (%)		
Recurrence	5 year	−	39	12(70.6)	6 (54.5)	8 (88.9)	0.244	0.0876
		+	18	5 (29.4)	5 (45.5)	1 (11.1)	0.801	‐
	10 year	−	36	12 (70.6)	5 (45.5)	6 (66.7)	0.228	0.0651
		+	21	5 (29.4)	6 (54.8)	3 (33.3)	1.103	‐
Metastasis	5 year	−	38	12 (70.6)	8 (72.7)	8 (88.9)	0.172	0.00212*
		+	19	5 (29.4)	3 (27.3)	1 (11.1)	1.313	‐
	10 year	−	35	12 (70.6)	7 (63.6)	7 (77.8)	0.169	0.00343*
		+	22	5 (29.4)	4 (36.4)	2 (22.2)	1.208	‐
Dead of disease	5 year	−	39	12 (70.6)	8 (66.7)	9 (100)	0.169	0.00034*
		+	18	5 (29.4)	4 (33.3)	0 (0)	1.854	‐
	10 year	−	34	12 (70.6)	5 (41.7)	9 (100)	0.169	0.00034*
		+	23	5 (29.4)	7 (58.3)	0 (0)	1.854	‐

At the 5‐year follow‐up, 18 patients had high‐grade soft tissue sarcomas (STS) with recurrence, 19 had metastasis, and 18 had died of disease. After 5 more years, three more patients had experienced a recurrence, three more patients showed metastasis, and five more patients had DOD. The top three pathological types for most patients were shown. *Based on the Mann–Whitney *U*‐test, the thrombomodulin (TM) levels of patients with metastasis and those who had died of disease were significantly higher. UPS, undifferentiated pleomorphic sarcoma

### *Receiver Operating Characteristic Analysis*

For ROC analysis, the diagnostic accuracy for identifying 5‐year and 10‐year DOD was evaluated with the AUC in high‐grade STS (5 years: AUC 0.798, 95% confidence interval (CI) 0.676–0.922, 10 years: AUC 0.765, 95% CI 0.632–0.897). With a threshold of 0.283, the sensitivity and specificity for identifying 5‐year DOD were 88.9% and 71.8%, respectively, and for identifying 10‐year DOD were 78.3% and 73.5%, respectively. To divide patients into two groups for further analysis, a threshold of 0.283 was adopted, and low (≤0.283) and high (0.283<) TM mRNA groups were analyzed.

### *Logistic Analysis*

Multivariate logistic regression analyses were performed to elucidate the association of multiple factors for identifying 10‐year recurrence, metastasis, or DOD. Only TM mRNA levels higher than 0.283 were associated with a significantly increased risk of recurrence, metastasis, and DOD (recurrence: odds ratio [OR] 4.23, 95% CI: 1.05–17.0, *P* = 0.041, metastasis: OR 9.80, 95% CI: 2.06–46.0, *h* = 0.0040, DOD: OR 9.34, 95% CI: 2.05–42.7, *P* = 0.0038) (Table 4).

**TABLE 4:** Multiple logistic analysis to identify recurrence, metastasis, or DOD is shown

Characteristics	10‐year recurrence			10‐year metastasis			10‐year DOD		
	OR	95%CI	*P*‐value	OR	95%CI	*P*‐value	OR	95%CI	*P*‐value
Male	0.77	0.22–2.71	0.686	0.78	0.20–2.97	0.720	2.22	0.55–8.94	0.260
Age	1.00	0.97–1.03	0.994	0.97	0.94–1.01	0.189	1.00	0.96–1.04	0.907
Size	0.93	0.82–1.06	0.320	1.01	0.88–1.15	0.872	1.01	0.88–1.15	0.870
Deep	0.82	0.11–5.95	0.847	1.42	0.16–12.4	0.750	3.43	0.32–63.6	0.259
Trunk	2.09	0.48–8.98	0.321	1.25	0.26–5.86	0.777	2.02	0.41–9.83	0.383
High TM	4.23	1.05–17.0	0.041*	9.80	2.06–46.0	0.0040*	9.34	2.05–42.7	0.0038*

*Only a high thrombomodulin (TM) value showed a significant difference for detection of recurrence, metastasis, and died of disease (DOD).

Bold values for significant data.

### *Kaplan–Meier* *Analysis*

**Fig. 1:**
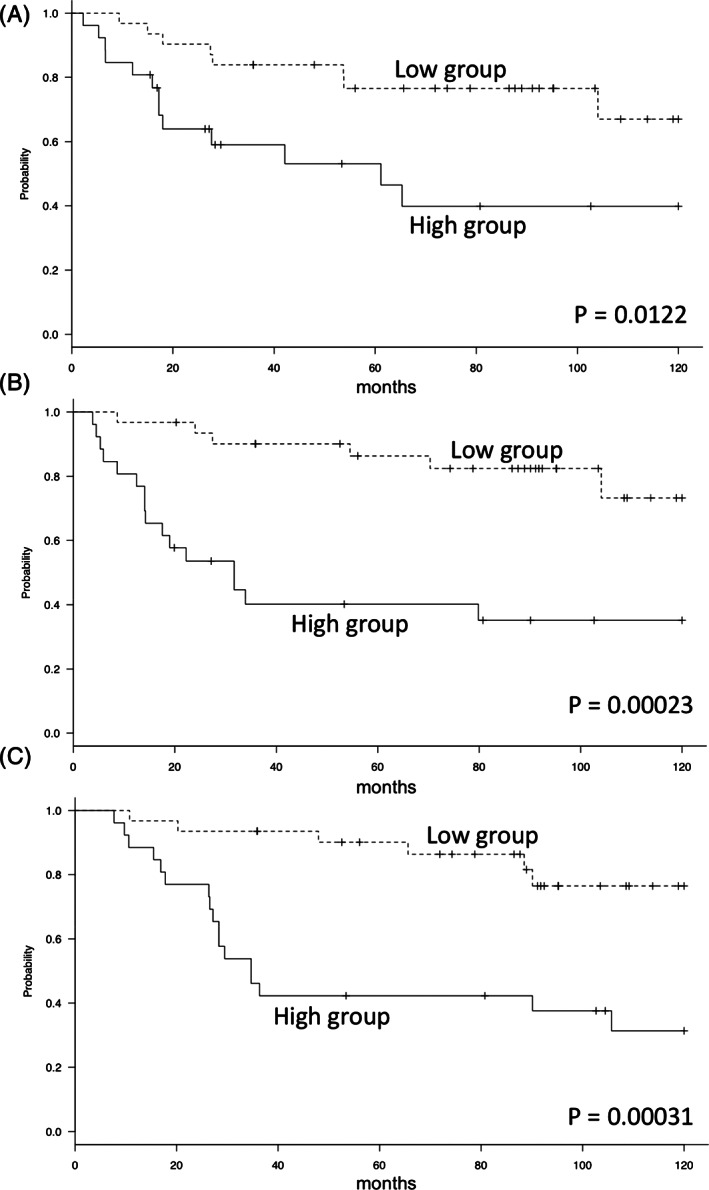
Kaplan–Meier curves in high‐grade soft tissue sarcoma (STS). The high thrombomodulin (TM) group had a significantly lower recurrence‐free survival (RFS) rate (5 years: low = 76.6%, high = 53.1%, 10 years: low: 67.0%, high 39.8%, *P* = 0.0122) (A). The high TM group had a significantly lower metastasis‐free survival (MFS) rate (5 years: low = 86.3%, high = 40.2%, 10 years: low: 73.3%, high: 35.2%, *P* = 0.00023) (B). The high TM group had a significantly worse overall survival (OS) rate (5 years: low = 90.1%, high = 42.3%, 10 years: low: 76.4%, high 31.3%, *P* = 0.00031) (C).

The RFS, MFS, and OS between the patients showing low and high TM mRNA were analyzed with Kaplan–Meier analysis and the log‐rank test. A significant difference between the two groups was seen for RFS (5 years: low = 76.6%, high = 53.1%, 10 years: low: 67.0%, high 39.8%, *P* = 0.0122; Fig. 1A) and MFS (5 years: low = 86.3%, high = 40.2%, 10 years: low: 73.3%, high: 35.2%, *P* = 0.00023; Fig. 1B). Furthermore, the high TM group showed significantly worse prognosis than the low TM group (5 years: low = 90.1%, high = 42.3%, 10 years: low: 76.4%, high 31.3%, *P* = 0.00031; Fig. 1C). Thus, high levels of TM mRNA are associated with highly recurrent and metastatic potential and lead to poor prognosis.

### *Cox Proportional Hazard Analysis*

The differences between the low and high TM mRNA groups were demonstrated with univariate Cox proportional hazard analysis. In RFS, only TM expression showed a significant difference (hazard ratio (HR): 2.95, 95% CI 1.21–7.19, *P* = 0.0168). In MFS, high TM and tumor size showed a significant difference (TM: HR: 4.97, 95% CI 1.93–12.8, *P* = 0.0009, size: HR: 1.08, 95% CI 1.01–1.17, *P* = 0.0258). In addition, to elucidate the association with multiple factors, multivariate Cox proportional hazard analysis was performed in MFS. Tumor size was not significantly different (*P* = 0.2961). High TM was the only independent metastatic factor (HR: 4.33, 95% CI 1.61–11.6, *P* = 0.00359). In OS, similar to MFS, not only high TM but also tumor size showed a significant association with the survival rate (TM: HR: 4.73, 95% CI 1.85–12.0, *P* = 0.0011, size: HR: 1.10, 95% CI 1.02–1.18, *P* = 0.0105). In OS, additional multivariate Cox proportional hazard analysis with high TM and tumor size showed a significant difference only for high TM (HR: 3.96, 95% CI 1.49–10.5, *P* = 0.00569) (Tables 5‐7).

**TABLE 5:** Univariate Cox proportional hazard analysis for recurrence‐free survival (RFS)

RFS	Univariate analysis		
	HR	95% CI	*P*‐value
Male	0.96	0.40–2.3	0.9324
Age	1.01	0.99–1.03	0.2614
Size	1.02	0.93–1.13	0.6640
Deep	0.64	0.18–2.21	0.4877
Trunk	1.53	0.56–4.20	0.4031
Radiation	1.35	0.57–3.22	0.4884
Chemotherapy	1.67	0.70–3.97	0.2444
High TM	2.95	1.21–7.19	0.0168*

In univariate analysis, only high TM showed a significant difference in RFS. TM, Thrombomodulin. CI, confidence interval; HR, hazard ratio.

**TABLE 6:** Univariate and multivariate Cox proportional hazard analysis for Metastasis‐free survival (MFS)

MFS	Univariate analysis			Multivariate analysis		
	HR	95% CI	*P*‐value	HR	95% CI	*P*‐value
Male	1.14	0.47–2.75	0.7546	‐	‐	‐
Age	1.00	0.98–1.02	0.7237	‐	‐	‐
Size	1.08	1.01–1.17	0.0258*	1.04	0.96–1.12	0.2961
Deep	1.25	0.29–5.39	0.7558	‐	‐	‐
Trunk	1.22	0.41–3.60	0.7195	‐	‐	‐
Radiation	1.47	0.59–3.61	0.4005	‐	‐	‐
Chemotherapy	1.54	0.65–3.62	0.3191	‐	‐	‐
High TM	4.97	1.93–12.8	0.0009*	4.33	1.61–11.6	0.00359*

In univariate analysis, for MFS and OS, size and high TM showed a significant difference. In multivariate analysis, only high TM showed a significant difference in MFS and OS. TM, Thrombomodulin. CI, confidence interval; HR, hazard ratio.

**TABLE 7:** Univariate and multivariate Cox proportional hazard analysis for Overall survival (OS)

OS	Univariate analysis			Multivariate analysis		
	HR	95% CI	*P*‐value	HR	95% CI	*P*‐value
Male	1.94	0.76–4.95	0.1633	‐	‐	‐
Age	1.01	0.99–1.03	0.2576	‐	‐	‐
Size	1.10	1.02–1.18	0.0105*	1.05	0.97–1.13	0.1548
Deep	2.57	0.34–19.2	0.3512	‐	‐	‐
Trunk	1.41	0.52–3.81	0.483	‐	‐	‐
Radiation	2.33	0.96–5.69	0.0614	‐	‐	‐
Chemotherapy	1.79	0.77–4.15	0.1710	‐	‐	‐
High TM	4.73	1.85–12.0	0.0011*	3.96	1.49–10.5	0.00569*

In univariate analysis, for MFS and OS, size and high TM showed a significant difference. In multivariate analysis, only high TM showed a significant difference in MFS and OS. CI, confidence interval; HR, hazard ratio; TM, thrombomodulin.

## Discussion

In experimental studies, TM protein has the potential to prevent metastasis, because of its anti‐coagulant activity^15^, ^18^. In various cancers, high expression of TM represents better prognosis than absent or low expression of TM^11^, ^16^, ^19^. However, we previously reported that high levels of soluble serum TM may be a significant predictor of metastasis and poor prognosis in STS patients^17^. In STS, the role of TM in tumor behavior may be different from that in other cancers.

In the current study, we found no difference in the level of TM mRNA between benign tumors and STS. In STS, no significant difference was seen between histological low‐grade and high‐grade tumors (Table 1). However, in high‐grade STS, TM levels in tumors over 10 cm were significantly higher than levels in tumors under 10 cm (Table 2). Furthermore, the threshold TM value of 0.283 was also useful for predicting 5‐year and 10‐year RFS, MFS, and OS. In Cox proportional hazard analysis, only high TM was a significant recurrent risk factor (HR: 2.95). According to univariate analysis, tumor size as well as high TM had the potential to indicate a risk of metastasis and poor prognosis. In multivariate analysis, only high TM indicated a significant risk of metastasis and poor prognosis (MFS, HR: 4.33, OS, HR: 3.96) (Table 5). Given this, measurement of TM mRNA was helpful for diagnosis and predicting recurrence, metastasis, and a poor outcome only in high‐grade STS. These results were similar to those of a previous study that showed that soluble TM was a poor prognostic indicator of STS^17^.

Thrombomodulin expression in STS cells is uncommon. Immunohistological staining for TM has been used as a marker of malignant mesothelioma^20^, and other studies have reported positive TM staining in angiosarcomas^21^, ^22^, ^23^. We speculate that the origin of TM mRNA in this study may not be STS cells but other cells. In this study, whole tumor tissues obtained by surgical resection or an open biopsy were analyzed without selection with microdissection techniques. Various cells, such as tumor cells, vascular endothelial cells, and fibroblasts, were included in the obtained tissues. Endothelial cells usually express TM, and TM is used as a marker of endothelial cells in histology. Amplified TM mRNA may have been derived not from tumor cells but endothelial cells. Histological analysis showed that vascular invasion is a significant prognostic factor for metastasis in STS^24^. TM mRNA in STS may correspond to vascular invasion in STS, which is a hypothesis that requires further study.

Translational medicine efficiently facilitates medical advances from the basic sciences to the clinical situation^25^. Measuring TM mRNA may be useful for identifying recurrence, metastasis, and poor prognosis in high‐grade STS. We believe that this translational study to identify and understand specific phenotypes of STS and its amassment will lead to improved sarcoma treatment.

This retrospective study has some limitations. The number of patients was small. Soft tissue tumors, including sarcomas, have many subtypes, and the incidence rate of each is low; because of the small number of patients, we could not statistically analyze the tumors by subtype. Many studies have analyzed STS as a whole group rather than by each histological classification^26^, ^27^, ^28^. In our previous study, we showed that soluble TM in serum is elevated in atrial fibrillation, organ failure, sepsis, disseminated intravascular coagulation, vasculitis, and venous thrombosis due to endothelial damage^17^. Circulating inflammatory factors may increase TM mRNA production in tumor tissues. These background conditions generally increase with age, and TM mRNA is thought to increase with age. Our univariate analysis did not show any relationship between poor MFS and OS in the high TM group or in older patients. These background conditions were not included in the statistical analysis in this study.

### *Conclusion*

The association between TM and STS is newly recognized. We successfully demonstrated the relationship between TM mRNA in STS tissues and recurrence, metastasis, and poor outcomes. We believe that the small step of this translational study will help increase the understanding of the mechanism of soft tissue tumor malignancy and accelerate drug development.
